# Benign ethnic neutropenia: an analysis of prevalence, timing and identification accuracy in two large inner-city NHS hospitals

**DOI:** 10.1186/s12888-021-03514-6

**Published:** 2021-10-13

**Authors:** Ebenezer Oloyede, Olubanke Dzahini, Nigel Barnes, Aleksandar Mijovic, Shreyans Gandhi, Sara Stuart-Smith, Theo de Witte, David Taylor, Eromona Whiskey

**Affiliations:** 1 Pharmacy Department, South London and Maudsley NHS Foundation Trust, London, UK; 2 King’s College London, Institute of Psychiatry, Psychology & Neuroscience, London, UK; 3 King’s College London, Institute of Pharmaceutical Science, London, UK; 4 Pharmacy Department, Birmingham and Solihull Mental Health NHS Foundation Trust, Birmingham, UK; 5 Kings College London NHS Foundation Trust, London, UK; 6 Radboud University Medical Center, Radboud University Nijmegen, Nijmegen, Netherlands

**Keywords:** Clozapine, Benign ethnic neutropenia, CNRD

## Abstract

***Background*:**

Benign ethnic neutropenia (BEN) is the most common cause of chronic neutropenia seen in individuals of African, Middle Eastern and West Indian descent. This phenotype is broadly defined by an absolute neutrophil counts (ANC) below 1.8 × 10^9^ cells/L in the absence of other causes, without an increased risk of infection. BEN has been implicated as a potential source of disparity in patients treated with clozapine, the antipsychotic of choice in treatment-resistant schizophrenia. Our main objective was to examine the current level of BEN recognition in a cohort of patients treated with clozapine and the potential impact of unidentified BEN on the initiation and maintenance of clozapine treatment.

***Methods*:**

This was an observational, retrospective analysis of patients registered with clozapine haematological monitoring systems in two large mental health trusts, chosen because they serve an ethnically diverse population. The first objective was to establish certified BEN prevalence in current users of clozapine. The second objective was to explore the stage of treatment at which BEN was identified. The third objective was to evaluate the extent of unrecognised BEN in patients registered on the Central Non-Rechallenge Database (CNRD), a database for patients whose haematological parameters fall below set thresholds when receiving clozapine treatment, meaning they cannot ordinarily be prescribed clozapine again.

***Results*:**

The study population comprised of 2020 patients on the clozapine register. 111 patients were monitored under BEN criteria. BEN was mostly identified after a below threshold haematological result or clozapine rechallenge (68%) compared to at clozapine initiation (32%). Eight of the 18 (42%) black patients registered on the CNRD were classified as BEN after assessment by a haematologist. Of these 8 patients, none would have met CNRD criteria again if monitored with BEN criteria at clozapine initiation.

***Conclusions*:**

Current evidence suggests that BEN remains an uncommonly recognised haematological phenotype. Improved timely identification of BEN will reduce unnecessary interruption or discontinuation of clozapine treatment. Our results suggest consideration should also be given to determining BEN status prior to initiating clozapine. Moreover, adoption of current FDA BEN monitoring criteria in the UK may further reduce clozapine discontinuation due to perceived neutropenia as drug toxicity, particularly in treatment-refractory schizophrenia patients.

**Supplementary Information:**

The online version contains supplementary material available at 10.1186/s12888-021-03514-6.

## Background

It has been recognised as early as the 1940s that individuals from certain ethnic populations, who are otherwise healthy and not prone to repeated or severe infections, commonly demonstrate recurrent low absolute neutrophil counts (ANC) below 1.8 × 10^9^ cells/L. [1] This phenomenon is commonly defined as benign ethnic neutropenia (BEN), or benign familial neutropenia, and has recently been linked to the atypical chemokine receptor 1 (ACKR1) gene, previously known as the Duffy antigen-negative phenotype produced by a homozygous state of DARC (Fy −/Fy-) gene [1–3]. Recent genetic studies support earlier reports stating that BEN most frequently occurs in individuals of African descent, with an estimated prevalence rate ranging from 25 to 50% [2]. Case report evidence also suggests its occurrence in some Caucasian and Chinese populations [4–8].

In the United Kingdom (UK), BEN is recognised as a modifier for the use of clozapine, the antipsychotic of choice for treatment-resistant schizophrenia (TRS) [9]. Since 2002, national guidelines provide separate algorithms for monitoring patients with and without BEN and establish a lower threshold for clozapine discontinuation for those with BEN (Table 1); however, little is known about how this has influenced clozapine prescriptions in the UK [2]. Supporting this criteria change were earlier large-scale epidemiological studies reporting unidentified BEN as a key reason for clozapine underutilisation and early discontinuation in the black and ethnic minority communities in the UK [10]. Despite this, some reports suggest that BEN remains under-recognised with clozapine use and that not all prescribers are sufficiently acquainted with this haematological phenotype and its management [2, 11–13].

**Table 1:**
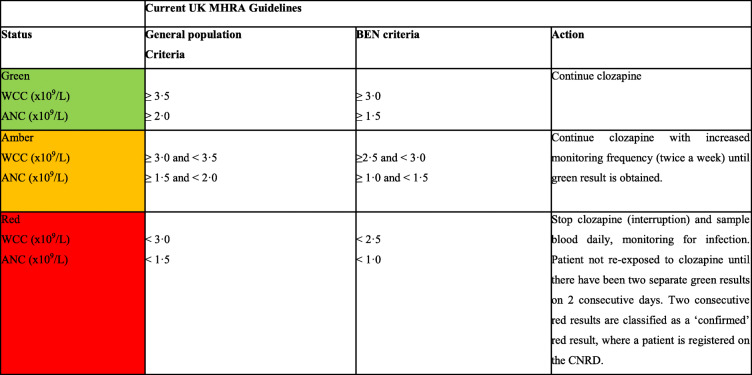
Summary of MHRA guidelines for clozapine monitoring in the UK

*ANC* Absolute Neutrophil Count, *BEN* Benign Ethnic Neutropenia, *CNRD* Clozapine Central Non-Rechallenge Database, *UK* United Kingdom, *MHRA* Medicines and Healthcare Products Regulatory Agency, *WCC* White Cell Counts

In recent years, there has been a concerted effort to improve clozapine use in TRS, reflecting an increased acknowledgement of its gross underutilisation, despite its well-documented therapeutic benefits [14]. A key aspect of this has been to achieve an optimal balance between the risks and benefits of mandatory haematological monitoring. A growing body of evidence suggests that the stringency of the monitoring for clozapine-induced blood dyscrasias, including agranulocytosis, may be disproportionate [15–18]. Our previous study on clozapine monitoring parameters in the UK found that there was a significant delay in the identification of BEN in a group of patients that were rechallenged on clozapine after a suspected haematological reaction. More specifically, in a third of the cases, BEN was only identified on rechallenge, suggesting a rather reactive approach to its recognition [19]. Similarly, earlier work in the same care setting demonstrated a relatively low identification of BEN in a group of patients treated with clozapine, as compared to documented prevalence rates [2].

Thus, the problems enumerated above suggest that the identification of BEN remains a clinical challenge and that timely identification of this benign phenotype could prevent avoidable clozapine discontinuation and ultimately improve clinical outcomes for many [19]. In this study, we attempted to investigate this hypothesis by evaluating current clinical practice and assessing the potential impact of unrecognised BEN for a cohort of patients restricted from clozapine use due to perceived haematological toxicity of the drug.

## Methods

We examined Zaponex Treatment Access System (ZTAS) and Denzapine Monitoring System (DMS) data for patients registered for clozapine use from South London and Maudsley (SLaM) NHS Foundation Trust and Birmingham and Solihull Mental Health NHS Foundation Trust (BSMHFT), respectively. SLaM provides mental health services to 1·2 million people across four South London boroughs whereas BSMHFT serves a population of 1.3 million in the West Midlands. These two Mental Health Trusts serve a population with high ethnic diversity. The first objective was to identify the proportion of patients registered with BEN who were actively being treated with clozapine at data collection. The second objective was to explore at what stage into treatment BEN was identified by a haematologist, where applicable. This was categorised as follows: on initiation, after a below threshold haematological result or on re-challenge with clozapine. The third objective was to identify potential cases of BEN in a group of black patients whose haematological parameters fell below certain thresholds and were placed on the Central Non-Rechallenge Database (CNRD). The CNRD is a mandatory database ensuring that patients are not inadvertently re-exposed to clozapine treatment, regardless of the pharmaceutical brand the patient has taken, is currently or may take in the future [19]. The number of black patients classified as having BEN who met CNRD criteria again despite BEN monitoring thresholds were also documented. The purpose of such analyses was to evaluate the impact of unidentified BEN on the initiation and maintenance of clozapine treatment.

### Definition of neutropenia, BEN and clozapine re-challenge

In the UK, clozapine monitoring is regulated by the Medicines and Healthcare Products Regulatory Agency (MHRA) using the criteria set out in Table 1, which includes lower haematological cut-off points for patients with BEN since 2002 [2]. To allow for this variation, the presence of BEN must be confirmed by a haematologist. In exceptional cases, clozapine manufacturers allow for monitoring under BEN criteria without formal identification under an off-licence agreement after a comprehensive evaluation of the risk and benefits, with input from a consultant haematologist. In the UK, there are three manufacturers of clozapine and associated haematological monitoring services; Clozaril® (Mylan) monitored by Clozaril Patient Monitoring Service (CPMS), Denzapine® (Britannia Pharmaceuticals Limited) monitored by DMS and Zaponex® (Leyden Delta BV) monitored by ZTAS [20–22].

In this study, haematological events were defined according to the MHRA guidelines for ANC and WBC values that required increased haematological monitoring, clozapine treatment interruption or discontinuation (Table 1). Under current recommendations, two consecutive red results are classified as a ‘confirmed’ red result. In this event, the relevant clozapine monitoring service submits the patient’s details to the CNRD, then the patient is classified as ‘non-rechallengeable’, where re-exposure to clozapine is not permitted, which is fully described elsewhere [19].

Clozapine manufacturers allow for the re-exposure of clozapine following CNRD registration under an off-licence agreement [19]. The decision for clozapine re-challenge is undertaken on an individual basis and must be agreed by a multidisciplinary team, in close liaison with a consultant haematologist. The final decision is driven by a comprehensive assessment, which includes extensive information gathered from various sources such as haematological profiling.

### Study sample

The study sample was identified within SLaM and BSMHFT on the 1st October 2020. Patients were identified using the monitoring database used in each trust: ZTAS and DMS respectively. Where applicable, the date of BEN identification was recorded from the ZTAS and DMS databases. Patients who were monitored via BEN criteria off-licence were also identified.

Demographic and prescription information at data collection were obtained from electronic medical records and dispensing records at SLaM and BSMHFT. Self-reported race was coded as follows: Asian, Black African/Caribbean, White and Other. Individuals of mixed race were coded as other. The duration of treatment from the most recent clozapine initiation date was recorded. The number of haematological abnormalities (red and amber results) prior to BEN identification were collected for all patients.

### Determining BEN in the CNRD cohort

SLaM patients recorded on the CNRD database were identified from ZTAS. The database was screened for black patients who had not been monitored under BEN criteria prior to CNRD registration. Black patients were selected as BEN is reported to occur predominantly in this population [1].

As recently demonstrated by Merz and colleagues, the number of neutrophils in individuals with BEN is perfectly adequate for host defence, so it should be considered a normal variant and not a “condition” or “disease” [23]. Duffy antigen phenotyping or ACKR1 gene sequencing is considered gold standard for identifying BEN, but this procedure is not currently performed in either of the study sites. Therefore, in the current study potential BEN was determined in two stages. At the first stage, at least two neutrophil counts prior to clozapine initiation, an ethnic origin enquiry and baseline blood tests including full blood count and film, routine biochemistry, autoantibody screen, virology and haematinics to exclude other pathology were sent to two independent haematologists who classified cases as either “BEN (B)”,“BEN excluded (BE)” or “BEN undetermined (BU)” using criteria recommended by Manu and colleagues [1]. This included assessing at least two full blood count readings at least 1 week apart where available. At the second stage, any cases in which the haematologists did not come to a consensus were sent to a third haematologist (blinded to the previous ruling) to aid in arbitration. Cases that could not be confidently confirmed or excluded as BEN following review by a haematologist remained in the third group of “BEN undetermined”. For those classified as BEN, UK BEN monitoring parameters were applied to previous haematological readings to determine whether CNRD registration would have occurred, had they been certified as BEN initially.

### Ethical approval

This study was approved by SLaM and BSMHFT Drug and Therapeutics Committees (Approval number: DTC/2020/115), the locally designated ethical approval committees for all noninterventional prescribing outcome evaluations, and the analysis used anonymised clinical data. Informed consent for the current study is waived by SLaM and BSMHFT Drug and Therapeutics Committee.

### Statistical analysis

Baseline demographics and clinical data were summarised using descriptive statistics. Means, standard deviations (SDs), medians and interquartile ranges were calculated for continuous data. Frequencies and percentages were calculated for categorical data. Interrater agreement between haematologists during the retrospective BEN classification process was assessed using Cohen’s kappa coefficient. Statistical analysis was performed using SPSS for Windows, version 27.

## Results

A total of 2020 patients had been registered on ZTAS and DMS. A total of 574 black patients (28%) were treated with clozapine at data collection. Of these, 444 patients (77%) were receiving care from SLaM and 130 (23%) from BSMHFT. 84 (19%) of these individuals were monitored under BEN criteria in SlaM and 16 (12%) in BSMHFT, equating to 17% of black patients overall (Supplementary appendix 1). A total of 1446 non-black patients (72%) were treated with clozapine at data collection. Of these, 717 patients (49%) were receiving care from SLaM and 729 (51%) from BSMHFT. 5 (0.7%) of these individuals were monitored under BEN criteria in SLaM and 6 (0.8%) in BSMHFT, equating to 0.8% of non-black patients overall. Clinical and demographic characteristics of the current users of clozapine monitored under BEN criteria are shown in Table 2.

**Table 2:** Socio-demographic and clinical characteristics of patients registered with BEN

Characteristic	SLaM (***n*** = 89)	BSMHFT (***n*** = 22)	Total (***n*** = 111)
**Male gender (%)**	60 (67)	19 (86)	79 (71)
**Age at BEN registration (mean years ± SD)**	35 (11)	34 (13)	35 (12)
**Duration of illness (mean years ± SD)**	16.6 (5.6)	ND	ND
**Time to clozapine initiation from illness onset**			
**(mean years ± SD)**	5.1 (4.5)	ND	ND
**(median years, IQR)**	4.0 (7.0)	ND	ND
**Time to BEN identification from first clozapine initiation**			
**(mean years ± SD)**	3.1 (4.3)	0.7 (1.4)^a^	2.7 (4.0)
**(median years, IQR)**	1.2 (4.0)	0.1 (0.7)^a^	0.6 (3.3)
**Diagnosis (%)**			
F20 Paranoid Schizophrenia	72 (82)	22 (100)	94 (84)
F25 Schizoaffective disorder	12 (13)	0 (0)	12 (11)
F31 Bipolar disorder	2 (2)	0 (0)	2 (2)
Other	3 (3)	0 (0)	3 (3)
**Psychiatric co-morbidity (%)**	5 (6)	0 (0)	5 (5)
**Ethnicity (%)**			
White	4 (4)	1 (5)	5 (5)
Black	84 (95)	16 (73)	100 (90)
Asian	0 (0)	0 (0)	0 (0)
Other	1 (1)	5 (22)	6 (5)
**BEN Identification (%)**			
By a haematologist	85 (96)	18 (82)	103 (93)
Monitored off-licence	4 (4)	4 (18)	8 (7)
**Red results prior to BEN identification**			
**n (%)**	28 (31)	6 (27)	34 (31)
**(mean, SD)**	1 (2)	1 (1)	1 (2)
**(median, IQR)**	0 (1)	0 (1)	0 (1)
**Amber results prior to BEN identification**			
**n (%)**	56 (63)	11 (50)	67 (60)
**(mean, SD)**	6 (10)	6 (12)	6 (10)
**(median, IQR)**	2 (6)	2 (6)	2 (6)

*BEN* Benign ethnic neutropenia, *BSMHFT* Birmingham and Solihull Mental Health NHS Foundation Trust, *IQR* Interquartile range, *SD* Standard deviation, *SLaM* South London and Maudsley (NHS Foundation Trust

^a^Missing data: 2 patients

### Treatment stage for BEN identification

Table 3 outlines and compares the treatment stage for when BEN was identified. 52% patients were identified with BEN only after a haematological abnormality was recorded with mandatory clozapine monitoring and another 16% after CNRD registration.

**Table 3:** Treatment stage of BEN diagnosis

Stage of BEN Identification	SLaM (***n*** = 89) n (%)	BSMHFT^**a**^ (***n*** = 17) n (%)	Total (***n*** = 106) n (%)
At Initiation	27 (30)	6 (35)	33 (32)
After Below Threshold Haematological Reaction	45 (51)	11 (65)	56 (52)
On Re-challenge	17 (19)	0 (0)	17 (16)

^a^Missing data: 5 patients

### Time to BEN identification

**Fig. 1:**
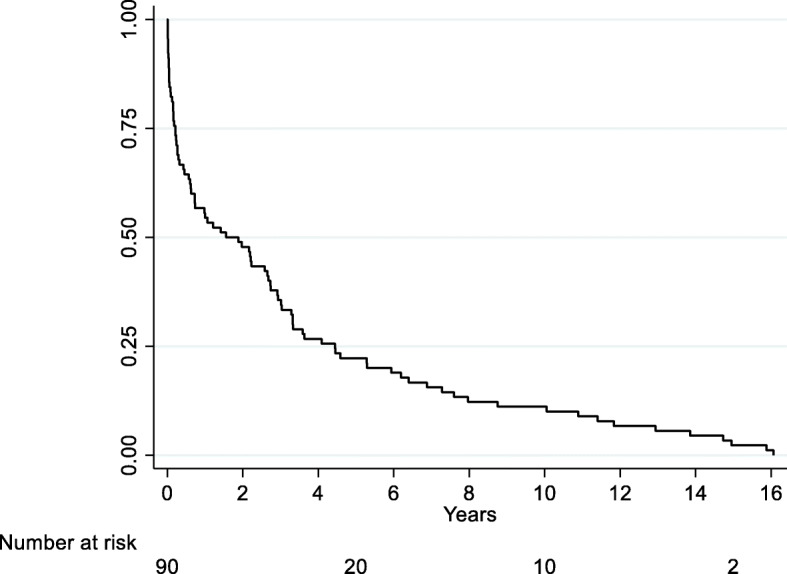
Kaplan Meier for the time to BEN registration from clozapine initiation

Figure 1 shows the Kaplan-Meier curve of the time to BEN registration from clozapine initiation. The median survival time to BEN registration from clozapine initiation was 1.0 (IQR 3.0) years.

### CNRD cohort

A total of 84 patients were registered on the CNRD at data collection. Of these, 69 patients (82%) were receiving care from SLaM and 15 (18%) from BSMHFT. 4 (6%) of these individuals were monitored under BEN criteria in SlaM and 3 (20%) in BSMHFT, equating to 0.7% overall.

26 black patients were registered on the CNRD without prior monitoring using the BEN criteria at SLaM. Of these, 18 patients had available haematological data for the retrospective BEN classification. After the classification process, 8 of the 18 suspected cases (44%) were identified as BEN, and 1 case (6%) remained undetermined. In the remaining 9 cases (50%), BEN was excluded. For the 8 patients classified as BEN, none would have met CNRD criteria if monitored under BEN criteria at clozapine initiation.

Haematologist agreement was highly concordant (83%) and the Kappa score was 0.71 indicating substantial agreement. In the 3 cases (17%) where haematologists differed in opinion, the discrepancy included the categories “BEN” and “BEN undetermined”. In two of the three cases where arbitration was needed, the third haematologist agreed with one of the two previous haematologists.

### ANC and WCC distribution

**Fig. 2:**
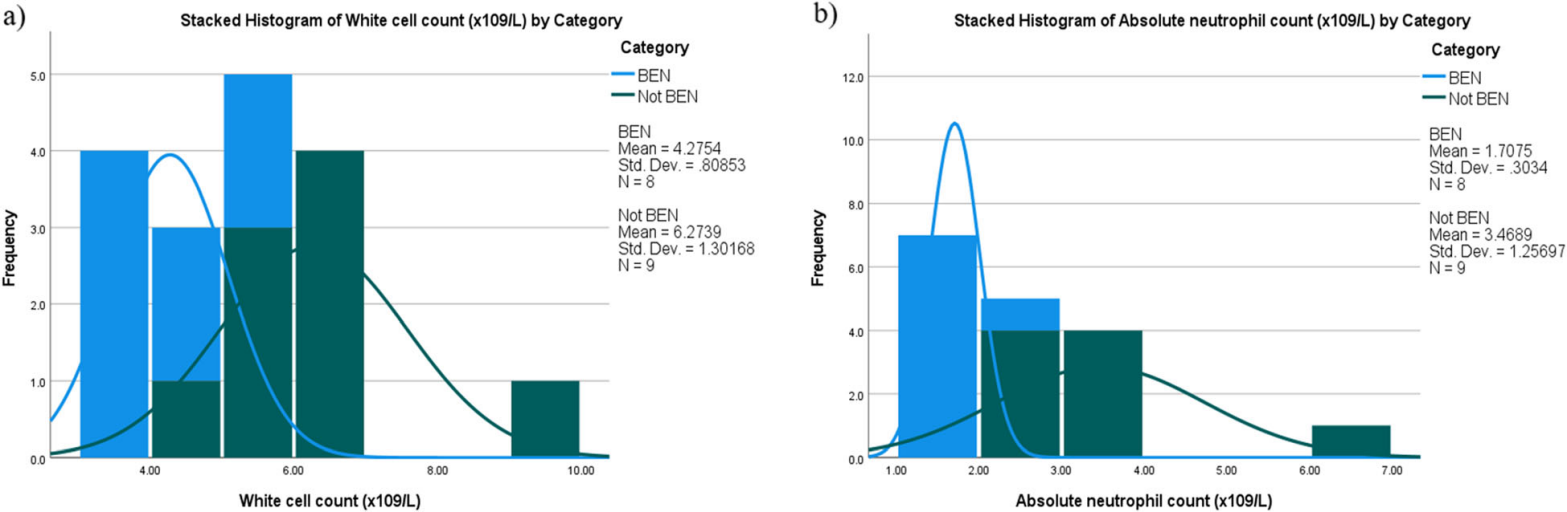
**a** Histogram of mean baseline WCC for those categorised as BEN (*n* = 8) and NOT BEN (*n* = 9) by haematologists (**b**) Histogram of mean baseline ANC for those categorised as BEN and NOT BEN by haematologists

Figure 2 shows the distribution of mean ANC and WBC prior to clozapine initiation for patients categorised as BEN and BEN excluded.

## Discussion

### Our findings

While the underutilisation of clozapine is a prevalent clinical challenge present in most countries, emerging evidence has suggested a greater occurrence amongst black patients, partly due to the under-recognition of BEN [24]. Here, we used a large retrospective cohort of patients treated in two large NHS hospitals to take a more detailed look at the clinical management of patients with BEN who are receiving clozapine treatment. We compared the findings to those described in the existing literature and identified the proportion of patients with unidentified BEN who had restricted access to this uniquely effective treatment.

In this study, we found that only 17% of black patients who were treated with clozapine were registered with BEN and subsequently monitored under the relevant criteria. Furthermore, for most of these patients (68%), registration with BEN occurred only after a previous below-threshold haematological result or on clozapine rechallenge. Moreover, our exploratory analysis demonstrated that 8 of 18 patients (44%) were restricted from using clozapine, which was likely due to unidentified BEN, as opposed to a true clozapine-induced neutropenia.

### Comparison with other studies

To our knowledge, this is the first study to demonstrate the implications of unidentified BEN on clozapine treatment maintenance for a cohort of patients with a refractory psychotic illness. The findings of the present study complement those of Whiskey and colleagues, where it was shown that only 8.4% of black patients who were treated with clozapine in one of the same care settings were registered with BEN [2]. While our findings indicate a twofold improvement in the recognition of BEN by clinicians in SLaM in recent years, our data reaffirms that BEN identification remains lower than the prevalence rates described in the literature suggesting a selection in practice [25].

### Variation in practice

In our study, we found a numerical difference between black patients who were identified with BEN across geographical locations. In SLaM, 19% of patients were identified with BEN while in contrast, only 12% of patients from BSMHFT were identified with BEN. This can be likely attributed to differences in clinical practice, especially as previous studies have highlighted clinician knowledge, experience, and confidence as a significant barrier to clozapine treatment [26]. For example, SLaM hosts several specialist services, including the National Psychosis Service, a tertiary referral service with a special interest in clozapine rechallenge for whom this is deemed to be safe, often in close liaison with other medical specialities such as haematology [27]. Recent geographical data has demonstrated a substantial variation in clozapine prescription rates in the UK, with suggestions for a national training programme for clinicians and the implementation of a ‘Hub and Spoke’ model to improve clozapine management practices [28–31].. While tentative, our data suggests that this variability in practice may also be present with the identification of BEN in those receiving clozapine treatment. Important efforts have been made to address this in the US, where clinicians are required to complete a brief assessment to ensure they understand BEN and its management prior to registering as a prescriber with the clozapine monitoring service [14]. Consideration should be given to implementing such requirements in the UK to provide added focus to haematological monitoring and increase prescriber confidence in identifying phenotypes such as BEN.

### Stage of BEN identification & red results after BEN

An important finding in this study was that 5268% patients were identified with BEN only after a haematological abnormality was recorded with mandatory clozapine monitoring and another 16% after CNRD registration. Furthermore, the mean time to BEN registration from clozapine initiation was 2.7 years (SD = 4.0). Despite BEN often being an incidental finding in routine clinical care [32, 33], our findings highlight the need for more definitive and precise guidelines for the screening of BEN in potential clozapine candidates, especially considering that BEN identification can be complicated in some individuals by periods of ANC readings within the widely accepted reference range [34]. In addition, the psychological impact of uncertainty experienced with clozapine interruption, continual monitoring and possible relapse cannot be overstated.

This is further emphasised by the average of one red and six amber readings, and the resulting number of days unnecessarily restricted from clozapine use in our cohort prior to BEN identification. From a practical perspective, timely identification of BEN for many of these patients would have pre-empted the burden of unwarranted additional haematological assessments and treatment interruption that are required in the event of an amber or red result. It is important to note that this has been reported as a significant reason for treatment discontinuation by patients [35]. Other recent studies have shown that abrupt discontinuation of clozapine can lead to severe withdrawal symptoms and there are suggestions of a reduced or delayed response to clozapine treatment after its discontinuation [36]. To date, there are no consensus guidelines in the UK for when BEN should be investigated in potential clozapine candidates. It is our opinion that assessments should be initiated at first contact with mental health services on a national level to reduce the risk of premature treatment discontinuation, if clozapine was later indicated [1, 37]. On a broader population level, if such conspicuous monitoring is developed as a national screening programme, this may improve patient outcomes in other clinical areas where racial disparities partly due to BEN have also been observed [38–41].

### CNRD

Figure 2 illustrates that a small but significant proportion of patients 8(42%) were proscribed from using clozapine, which was likely due to unidentified BEN, as opposed to our original suspicions of clozapine-induced neutropenia. Furthermore, it is important to note that none of the patients where BEN was ruled out were classified as true clozapine-induced agranulocytosis. This was also emphasised by all ANC readings being above the widely accepted 0.5 × 10 [9]/L threshold (results not shown). Differential diagnoses by haematologists included transient neutropenia and iron deficiency. Early recognition of BEN in these patients could have prevented initial CNRD registration and avoided premature treatment discontinuation and the often-associated marked negative impact on the clinical status with non-clozapine treatment strategies.

At present, approximately 4000 patients are currently registered on the CNRD in the UK, of whom 500 are estimated to be black and not diagnosed with BEN [19]. Extrapolating the results of our study may suggest that up to 210 patients could have continued clozapine treatment if BEN were identified on initiation of the treatment. However, further work is required to establish this, especially as it is unknown how many of these patients underwent past clinical investigations for BEN.

### BEN & clozapine monitoring guidelines

Neutrophil granulocytes are the most abundant circulating leukocytes in humans and play a key role in the defence against bacterial and fungal infection [26, 35]. The risk of clozapine-induced neutropenia is well-documented, with an estimated prevalence of 1–3% [36]. Nevertheless, this estimate is likely to be confounded by other differentials such as BEN [19]. In the second half of the twentieth century, neutropenia was defined as an ANC value less than 1500/μL in individuals after the age of one [28], statistically determined as two standard deviations below the population mean at the time [27]. However, this classification system was originally developed in a largely Caucasian population and has consequently been recognised to preclude those with haematological variants such as BEN [27, 37]. Such evidence led to the amending of clozapine guidelines in 2002 in the UK, which permitted more patients with BEN to have access to clozapine [1].

Furthermore, in 2015, the United States Food and Drug Administration adjusted the ANC threshold for interrupting and discontinuing clozapine in BEN patients such that clozapine is held when ANC falls below 500 μL, whereas in other countries it is held when ANC falls below 1000 μL [19]. In addition, the requirements for monitoring WCC were removed. This change was intended to allow more patients with BEN to be placed on clozapine when indicated [42]. Such changes have not been implemented in the UK (Table 1). As demonstrated by a recent study [19], changes in the US underscore the possibility that modification of prescription recommendations for clozapine in the UK may reduce racial disparities and encourage greater use of clozapine in populations with high BEN prevalence [42]. In contrast to the UK, updated US guidelines do not mandate haematology input for BEN identification and allow prescribers to indicate the presence of BEN with no strict definition when enrolling patients [14]. Though this may pose a risk of overestimating BEN prevalence, an optimal balance between the risks and benefits may be achieved by relaxing the monitoring requirements in the UK to ease access to clozapine and reduce unnecessary interruptions in treatment [43].

### Limitations & future studies

An arguable limitation of this study is the arbitrariness in our definition of ethnicity. While BEN is not difficult to identify in individuals from ethnic groups where BEN is established, the diagnosis becomes more complex and imprecise in countries such as the UK, where a high frequency of inter-ethnic admixture exists [44].. Furthermore, our study is limited by only including current users of clozapine, as some may have stopped treatment prior to CNRD registration due to repeated testing. Nevertheless, our method is consistent with existing literature and may possibly even enhance our findings if BEN prevalence is higher when patients from a wider demographic are included. Ideally, future studies should look at pharmacogenetic testing to identify the ACKR1 genotype (rs2814778 C-C allele SNP) opposed to self-declared ethnicity when screening for BEN [3, 23, 45, 46] especially considering that studies have demonstrated a weak correlation between skin colour or self-declared race and genetic ancestry [47]. While diagnostic agreement between haematologists was very good in our study, the investigational emphasis relies on excluding other putative causes for neutropenia, and entails time-consuming, laborious and expensive tests, to make a diagnosis of exclusion, for essentially a non-pathogenic ethnic variation in normality. However, further work is required to validate a genetic test and to determine its cost-effectiveness and patient acceptability in routine clinical practice.

The existing study is also limited by the lack of consensus around the prevalence rate of BEN. While most investigators broadly describe BEN prevalence in 25–50% of black people [2], some studies report lower rates in similar populations, even with different geographic regions in the African continent and the middle-east. (However, this is possibly related to the presence of Caucasian heritage in patients classified as “Black” in these studies, resulting in heterozygosity for the ACKR1 genotype) [1, 48, 49]. Notwithstanding, the proportion of patients identified with BEN in our populations were still less than the lowest commonly reported prevalence rate.

While a growing body of literature exists on the pathophysiology of BEN [45, 50, 51], authors have noted that ANC reference ranges in many countries are not representative of the entire population [23] and some have suggested the need for ethnic-specific reference ranges for haematological values to reduce racial disparities in patient outcomes [52, 53]. However, this is further complicated by reports of more than one reference range for haematological values in a single ethnic or racial group [54, 55]. Future work should look to determine the feasibility of ethnic-specific reference ranges based on genetics for neutrophil counts in those with BEN and the relevant cut-off values that ensure patient safety. Such genotyping studies may certainly help in clarifying borderline neutrophil counts, however in its present state, they can be time-consuming and expensive, moreover the full range of mutational variants seen in BEN have not been completely elucidated. In instances, they require the help of a biostatistician with expertise in variant calling, as well as other in-silico prediction tools/software, where it is a variant of uncertain significance. Notwithstanding, Duffy antigen testing provides a quicker and likely less expensive alternative, particularly if implemented into routine practice [46, 49].

## Conclusion

The current evidence suggests that BEN remains an underdiagnosed haematological phenotype. Better identification of BEN will reduce unnecessary interruptions or the discontinuation of clozapine treatment, along with the associated deterioration in mental state. Moreover, implementation of updated FDA BEN monitoring criteria, better awareness within the specialty and multi-disciplinary input, including from the pharmacist and haematology teams, in the UK may further reduce inappropriate clozapine discontinuation due to perceived haematological toxicity. Future developments will likely see the incorporation of genotyping studies, and better-defined criteria, in categorising BEN.

## Supplementary Information

**Additional file 1.**

## Data Availability

The anonymised datasets used and analysed during the current study available from the corresponding author on reasonable request.
