# Supplementary material for: Benign ethnic neutropenia: an analysis of prevalence, timing and identification accuracy in two large inner-city NHS hospitals
Source: BMC Psychiatry. 2021 Oct 13;21:502. doi: 10.1186/s12888-021-03514-6 (PMC8515765; doi:10.1186/s12888-021-03514-6)
Supplement: Supplementary file 1 — Additional file 1. [file 12888_2021_3514_MOESM1_ESM.docx]

Study profile
